# Incorporating Artificial Intelligence in Qualitative Research: Exploring the Role of ChatGPT in Thematic Analysis

**DOI:** 10.1007/s40670-025-02587-2

**Published:** 2025-12-04

**Authors:** Jonathan Bowden, Megha Mohanakrishnan, Andrew R. Thompson

**Affiliations:** Department of Medical Education, University of Cincinnati College of Medicine, 231 Albert Sabin Way, ML 0667, Cincinnati, OH 45267 USA

**Keywords:** AI, Generative AI, ChatGPT, AI in medical education, Medical education, Thematic analysis, Qualitative research

## Abstract

Thematic analysis is a form of qualitive analysis performed to identify patterns within text-based datasets such as open-ended responses. While thematic analysis is used extensively in medical education research, it has several limitations, such as subjective interpretation by graders and the time required to manually code responses in large datasets. There is potential to overcome many of these challenges with the use of Artificial Intelligence (AI) platforms, such as the free to use large language model, ChatGPT. The goal of this study was to evaluate whether AI can be used in thematic analysis to replace manual graders as the gold standard. The dataset used in this study was related to first year medical students’ thoughts and feelings regarding the act of cadaveric dissections. Three different methods were used to instruct the AI to grade the responses, and each method was repeated three times. Various measures related to precision and accuracy were compared, both within the repeated tests using AI and between the AI generated results and those obtained by manual coders. Results show that Method 3 had greater accuracy and agreement with the manual coders, but less precision compared to the other two methods. All methods had an agreement greater than 80%. These findings demonstrate that AI has promise in being used for thematic analysis, but the method used to instruct the AI has a strong influence on the results. When using AI for thematic analyses, it is imperative to carefully document and refine the methodology as well as still incorporate some human oversight to ensure accurate results.

**Supplementary Information:**

The online version contains supplementary material available at 10.1007/s40670-025-02587-2.

## Introduction

 Although qualitative analysis is a powerful tool in medical education research, it can be labor intensive when large amounts of data are involved. A commonly used method in qualitative research is thematic analysis, which aims to identify patterns or themes within a dataset [1]. Datasets are often generated from study participant responses to open-ended prompts or questions. Themes are either identified by the inductive approach, which derives themes from patterns found in the data, or the deductive approach utilizes pre-existing theories to form the themes independent of what is found in the data [2]. However, to compile the data for analysis, researchers must manually analyze the responses so that various codes and/or grading scales can be assigned to the participant responses. Without taking the necessary steps to reduce subjectivity that is inherent to this approach, inter-coder variation can introduce potential biases into the results [3].

In recent years, advancements in Artificial Intelligence (AI) has sparked interest in its application within medicine and medical education [4, 5]. Some examples that have been observed include enhancing virtual, visual simulations [6] and documenting notes for the electronic medical records [7]. While there are many types and subsets of Artificial Intelligence such as generative AI, agentic AI, and explainable AI [8, 9], an area of particular interest is the potential use of large language models in AI, where the program can generate human language or classify text, otherwise known as natural language processing tasks [10]. One of the most widely known examples of a large language model is ChatGPT, developed by Open AI. ChatGPT is an example of deep learning, which is a subset of machine learning type of AI [11]. Deep learning utilizes multiple hidden layers of neural networks to train its model, which was based on the understanding of neural biology [12]. In the example of ChatGPT, its model is the Generative Pre-trained Transformer (GPT), which uses deep learning to analyze large amounts of data such as news articles, books, and websites to learn patterns in text and help generate text of its own [13].

Since ChatGPT has a no cost option and is simple to use with the model being able to understand language commands without any specific syntax or coding, many have focused on the potential roles it has in medical education such as generating multiple choice questions [14], assessing the grammar of assignments [15], and generating interactive patient scenarios [16].

Given recent advancements and widespread availability of AI platforms, there is great potential to overcome many of the limitations surrounding efficiency and subjectivity involved with thematic analysis. Therefore, the goal of this study is to explore the role of AI in medical education research by comparing its performance to those done by manual graders when conducting thematic analyses.

## Materials and Methods

### Data Collection

This study utilized a dataset of open-ended responses from first-year medical students who were given the prompt, “please create an image of any sort relating to your thoughts or feelings as you anticipate the experience of dissection of a human body,” as well as to provide, “an explanation of your drawing or image.” Inductive thematic analysis was applied to the explanations to determine if there were any common themes in the response data. Themes were created by utilizing an initial sample of 30 responses randomly selected from the dataset. Initially, the 30 responses were submitted to ChatGPT several times, which was prompted to find the most recurring themes without specific guidelines to the number of themes to allow variation in responses. After numerous iterations, authors JB and MM compiled the different results, compared the themes to each other and the 30 responses to ensure additional themes were not missed, and made a final list of 14 themes. After the themes were created with accompanying definitions, an interobserver error study was performed where 20 of the responses were coded by both observers (JB and MM). After obtaining an agreement of > 90%, the final 14 themes and their definitions were refined and finalized based on discussion between the observers (Supplemental Table 1). Following this process, authors JB and MM each manually coded half of the dataset (combined total of *N* = 343 responses) using the final set of 14 themes. This study was approved by the University of Cincinnati IRB (#2023 − 0814).

### Thematic Analysis Using ChatGPT

The experiment used ChatGPT version 3.5 as the source of AI because of its wide use and open access without subscription. All 343 responses were submitted to ChatGPT using three different prompting methods. This was done to determine if the way AI was instructed had an impact on the results. **Method 1** of instructing ChatGPT was the most basic and entailed asking it to code the responses using only the list of themes and their definitions. **Method 2** added to Method 1 by providing 25 example responses, along with the themes assigned by the authors (JB and MM) with a brief explanation of why a given theme was assigned and instructed to utilize the examples as a reference for future coding. Finally, **Method 3**, like Method 2, provided 25 example responses, but in this case the AI was instructed to code one example response in a single prompt. ChatGPT was then provided with an explanation of why a given theme was either incorrectly assigned or missing and was asked to create a modified definition for the themes incorrectly coded to reflect the explanation; the theme definitions would be modified each time ChatGPT would be inconsistent with the human coders in the sample 25 responses. After performing this process for all 25 example responses, ChatGPT then graded the remaining responses using the edited definitions of the themes. While the methodology and amount of information given to the AI was different, the style of entering the prompts was kept consistent throughout the experiment to limit any confounding variables. While there is no standardized way of drafting a prompt to command ChatGPT for a task, there are many methodologies such as the “RISEN Framework” found in the article *Exploring Different Prompt Frameworks and Their Applications* that have certain steps to ensure the prompts were consistent and clear [17]. Using this framework, prompts would define the role the AI would take (researcher in qualitative analysis) with clear instructions and steps that it would take to achieve a defined end goal (i.e. when listing the prompts, create a table that will have the response in one column and the titles of the themes for the corresponding prompt).

### Evaluation of Accuracy and Precision

**Fig. 1:**
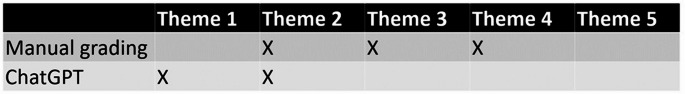
Example of the comparison of grading between the manual graders and ChatGPT to show the total and marked accuracy calculations. In this scenario, theme 2 is the only theme marked by both graders and theme 5 was the only theme not to be marked by both graders. This would give it a total accuracy of 2/5, and since the manual graders had a total of 3 themes found, this would have a marked accuracy of 1/3

Conventionally, accuracy is the closeness of a measured score to the true value, and precision is the agreement of repeated measurements [18]. Accuracy of the AI scoring was calculated by comparing its assignment of themes (coding) to those done by the manual graders. Accuracy was measured using two different criteria. The first criteria, referred to as “total accuracy”, viewed accuracy as the AI correctly assigning a code or correctly *not* assigning a code to each response. The second criteria, called “marked accuracy”, considered accuracy only in terms of correspondence of codes assigned by the authors and those determined by AI. An example of the differences in these forms of accuracy is displayed in Fig. 1. Each method of instructing ChatGPT was repeated three times. This was done to compare the intra-observer error of ChatGPT for both total precision and marked precision, similar to that of accuracy.

### Statistical Methods

ANOVA on Ranks was used to determine if any specific methodology had greater accuracy or precision compared to the others. Cohen’s kappa was used to determine the agreement between AI generated vs. manual coding [19]. Finally, Marascuilo’s Procedure was used to determine differences between each methods’ ability to identify certain themes. Accuracy between the different methods and between the different themes as well as agreement between AI and manual graders was tested using only the first results of each method. Alpha value was set at 0.05 with false discover rate to account for the family-wide error rate. All statistical analyses were performed with SigmaPlot and figures were generated using Microsoft Excel.

## Results

### Comparison Between Methods of AI Instruction

**Fig. 2:**
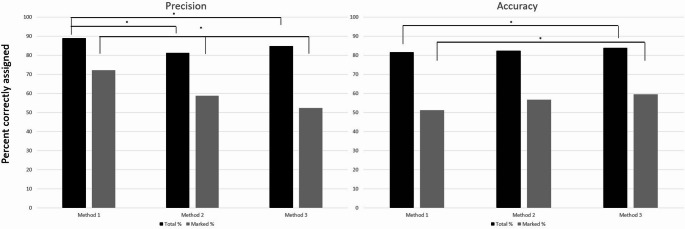
Differences between the three different methods of instructing ChatGPT for Precision (**A**) and Accuracy (**B**). For total precision and accuracy, Method 1 was more precise than Method 2 (*p* < 0.01) and Method 3 (*p* < 0.01), but Method 3 was more accurate than Method 1 (*p* = 0.26). For marked measurements, all methods differed in precision (*p* < 0.01), and Method 3 was more accurate than Method 1 (*p*< 0.01)

The different methodologies of instructing ChatGPT varied across their precision and accuracy. Figure 2A shows that the total precision was fairly consistent with only Method 1, with a precision of 89.0%, being greater than the total precision for Method 2 (81.2%, *p* < 0.01) and Method 3 (84.7%, *p* < 0.01); however, marked precisions were different across all the methods with Method 1 having a marked precision of 72.2%, Method 2 with 58.7%, and Method 3 with 52.3% (*p* < 0.01 across all comparisons). Figure 2B shows a similar trend with an increase in total accuracy between Methods 1 (81.7%) and Method 3 (83.0%, *p* = 0.026) as well as an increase in marked accuracy between Method 1 (51.2%) and Method 3 (59.6%, *p* < 0.01).

### Comparisons Between Human Graders and AI

Agreement between codes assigned by authors JB and MM and those determined by AI are shown in Table 1. Overall, the results were fairly similar with a moderate agreement for Method 1 (81.7%; Cohen’s k: 0.43), Method 2 (82.2%; Cohen’s k: 0.47), and Method 3 (83.9%; Cohen’s k: 0.50). Furthermore, all methods displayed a similar pattern of poor sensitivity (< 60%) but strong specificity (> 85%).

**Table 1:** Comparisons of the three methods of AI thematic analysis for total accuracy with manual grading in respect to all the themes that were marked across all prompts. The tables display the total number of themes that were included by both graders, one grader, or none of the graders. A) Method 1 had an 81.7 % agreement with moderate agreement (Cohen’s k: 0.43). B) Method 2 had an 82.2% agreement with moderate agreement (Cohen’s k: 0.47). C) Method 3 had an 83.9% agreement with moderate agreement (Cohen’s k: 0.50). All methods display a poor sensitivity (<60%) but a strong specificity (>85%)

	Manual Marked	Manual Not Marked
Method 1		
AI Marked	536	370
AI Not Marked	511	3385
Method 2		
AI Marked	558	374
AI Not Marked	429	3161
Method 3		
AI Marked	555	294
AI Not Marked	432	3241

### Comparisons Between AI Marked Accuracy By Individual Theme

**Fig. 3:**
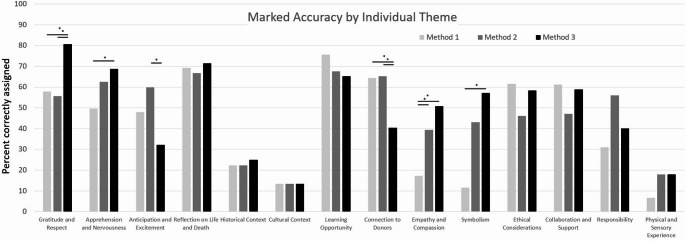
Comparisons of the three methods of AI marked accuracy based on the different themes. There were 14 different themes total that could be identified, and we looked at AI being able to detect the theme compared to the manual grading. Furthermore, the different methods also performed differently on certain themes. Method 3 was able to have a higher accuracy than Method 1 for the themes: “Gratitude and Respect” (*p* < 0.01), “Apprehension and Nervousness” (*p* = 0.01), “Empathy and Compassion” (*p* < 0.01), and “Symbolism” (*p* < 0.01). Method 3 was also more accurate than Method 2 for the theme “Gratitude and Respect” (*p* < 0.01) but was less accurate than Method 2 for “Anticipation and Excitement” (*p* < 0.01) and “Connection to Donors” (*p* < 0.01). Finally, Method 1 was more accurate than Method 3 for the theme “Connections to Donors” (*p* < 0.01) but was less accurate than Method 2 for the theme “Empathy and Compassion” (*p* = 0.01)

The marked accuracy scores for each methodology of instructing AI for all 14 themes differed both in performances based on the methodology and the specific theme being tested (Fig. 3). Method 3 had a higher accuracy than Method 1 for the themes “Gratitude and Respect” (80.6% vs. 57.9%; *p* < 0.01), “Apprehension and Nervousness” (68.8% vs. 49.6%; *p* = 0.01), “Empathy and Compassion” (50.7% vs. 17.3%; *p* < 0.01), and “Symbolism” (50.7% vs 11.5%; *p* < 0.01). Method 3 was also more accurate than Method 2 for the theme “Gratitude and Respect” (80.6% vs 55.6%; *p* < 0.01) but was less accurate than Method 2 for “Anticipation and Excitement” (32.1% vs. 59.9%; *p* < 0.01) and “Connection to Donors” (40.4% vs 65.2%; *p* < 0.01). Finally, Method 1 was more accurate than Method 3 for the theme “Connections to Donors” (64.4% vs 40.4%; *p* < 0.01) but was less accurate than Method 2 for the theme “Empathy and Compassion” (17.3% vs. 50.7%; *p* = 0.01).

## Discussion

Comparisons between the methodology of instructing ChatGPT underscores how results can be influenced by the manner in which the AI is prompted. Results from Methods 1 and 3 highlight this well, showing considerable difference in terms of precision and accuracy. Method 1 had a greater level of both total and marked precision than Method 3, but Method 3 had a greater total and marked accuracy compared to Method 1 (*p* < 0.05 for all comparisons). This may be because Method 1 had the most simplistic instructions where the AI only received definitions of the themes at each attempt. This could explain why its results were similar with each variation but were not as accurate. However, Method 3 was modeled based on how machine learning works by allowing the AI to have multiple attempts of analysis, which are then assessed and delivered back to allow the AI to “learn from experience”. Through these multiple iterations, the AI analysis may become more similar to that of the human graders and thus lead to a greater score in accuracy; however, since each iteration involves multiple dynamic interactions, its final results may vary substantially, which could explain the lower precision compared to Method 1.

Across all methods of instructing the AI, ChatGPT and the manual graders had agreement above 80%. While this did not reach the > 90% agreement that was achieved in the manual inter-observer error study, that does not necessarily mean it is inferior to the point of having no utility. While there is no standard accepted level of agreement for intercoder reliability, there are some recommendations such as the “rule of thumb” cited by Neuendorf where scores over 90% are acceptable by all, and scores over 0.8 are acceptable by many [20, 21]. There are also the recommendations by Landis and Koch where the scores 0–20% are slight, 21%−40% are fair, 41%−60% are moderate, 61–80% are substantial, and 81–100% are nearly perfect agreement [21, 22]. It must be emphasized that these scores are arbitrary in value, but from these guidelines, an argument can be made that utilizing AI for thematic analysis may already be ready or at least nearly ready to meet the standards that are currently in place, as long as the instruction methodology is carefully selected and deliberately implemented.

Finally, through the comparisons of individual themes, the results reinforce the potential benefit of instructing AI through Method 3, as it had the greatest number of themes with the highest grading accuracy. This may be due to the previously mentioned idea that having multiple interactions with the AI allows it to go through a learning process and further refine the internal definitions of each individual theme. However, even Method 3 had specific themes that performed low such as the themes of “Anticipation and Excitement” as well as “Connection to Donors”, having a marked accuracy of 32.1% and 40.4% respectively. One potential cause of this could be how the feedback was given or the lack of strong examples when going through the interaction phase with the AI. Finally, the themes “Historical Context”, “Cultural Context”, and “Physical and Sensory Experience” scored a low marked accuracy across all methods with the highest marked accuracy score being 25.0%, 13.3%, and 17.9% respectively. There are several potential reasons why these scores were lower, but the most likely issue is the low sample size of responses that were coded to these themes. Since marked accuracy is calculated using the number of responses coded by AI that correctly matched that of the responses coded by the manual graders for that theme, the score inherently depends on the number of responses where the manual graders assigned a certain theme. If a particular theme had more responses assigned to it, there was more opportunity for the AI to also code it correctly. The three themes previously mentioned only had 8, 15, and 28 coded responses, respectively, which could explain the lower accuracy. However, this may not be the only issue as other themes such as “Collaboration and Support” were coded for only 18 responses but still had a higher level of agreement of 58.8%. Another possibility could be the level of nuance in the theme. If this is the case, it may be more difficult for the AI to detect references to a historical or cultural event or, perhaps, the descriptions given for these themes were not adequate for the AI to identify these themes in a particular response.

### Limitations

This study has several limitations. The first is the version of AI that was used, ChatGPT version 3.5. While there is nothing inherently flawed about this version, AI is constantly evolving with newer models and versions being released. Currently, ChatGPT 4.0 is public and 4.5 available to select users with improved features such as scaling unsupervised reasoning, which improves the model’s reliability on topics [23]. Features such as these could potentially improve AI’s ability to perform thematic analysis and yield results varying from those found here.

Another limitation was the initial study design of forming the themes in the thematic analysis. The themes were formed based on a random sample of 30 responses, but these may not have been representative of all responses, which could leave prominent themes left unidentified. However, while this is a limitation of the thematic analysis, it is unlikely this would greatly impact on the purpose of this study, which was only validating AI’s ability to identify given themes in a dataset. Additionally, utilizing ChatGPT to identify the themes may have placed bias in the validation of the AI coding the themes, as these themes were created with assistance from ChatGPT, which may be more identifiable to AI compared to more complex human-generated themes. This is mitigated slightly, however, as the graders modified the list of themes and added and/or changed themes that were not listed by ChatGPT. Further research may benefit the credibility of the themes generated by ChatGPT through comparing the AI-generate themes with the intended message portrayed by the writers of the responses. This was unable to be achieved in this study as the data was anonymized. Finally, the refinement of the themes was done by the graders revising the themes generated by ChatGPT. However, future studies may reveal benefits of involving AI in the refinement of said themes could improve agreement of thematic coding between human and AI.

A third limitation is the generalization of AI’s ability to perform thematic analysis based on one specific dataset. Thematic analysis can be applied in many fields and assess a variety of potential topics. While this study specifically looked at emotional/attitude-related themes in open ended responses about the topic of cadaveric dissection, other thematic analyses may look at different themes focused on other topics and with texts in different formats, which may not reflect similar results. Furthermore, our study mainly focused on AI’s ability to perform a deductive thematic analysis where the AI was tasked to code the responses using a set of predefined themes. While the study did perform an inductive thematic analysis, where the themes were created based on repetitive patterns found in multiple texts, the effectiveness of the AI on this task was not explored. Finally, the study lacked any analyses on human level of precision compared to AI as well as the potential benefit of time-efficiency of AI compared to human. However, other studies have shown promise in both inductive performance and time-saving benefits in different topics [24, 25].

## Conclusion

Krippendorf [26] identifies that three aspects of coding go into the concept of reliability for thematic analysis: (1) stability – how coder behavior changes over time; (2) reproducibility – how multiple coders report differently; and (3) accuracy – how the coding properly analysis what it is supposed to look at [27]. This study explored how ChatGPT could be utilized for thematic analysis, using a dataset of medical student responses of their reflection on cadaveric dissection. The AI analysis shows an agreement with the human graders of greater than 80% across all trials, which others have argued can be viewed as a strong level agreement [20, 22]. Furthermore, it was shown that the method of prompting ChatGPT has a considerable impact on the results, with the largest difference related to the amount of instruction AI was provided during the analysis.

While there are several limitations, this study demonstrates the potential for AI to overcome some of the common pitfalls that researchers face when conducting a thematic analysis. However, it is not as simple as inputting a dataset into an AI platform, giving it a simple prompt, and getting back accurate and reliable results. As demonstrated here, there is still a considerable amount of planning and human interaction that must take place to produce reliable results using AI for thematic analysis. Further work like this study is needed to compare various versions of ChatGPT or different AI models as well as methodology of prompting AI to improve AI-assisted thematic analysis. This study, like many others, continues to advance the current understanding and potential of how large language model AI can be utilized in medical education. While it is important to continue to keep up with the technological advancements and implementation of these tools, it is equally imperative to determine the efficacy and limitations of these tools to understand how best to incorporate them into daily practice.

## Supplementary Information

Below is the link to the electronic supplementary material.

Supplementary File 1 (DOCX 15.4 KB)

## Data Availability

Data used in this study are available upon request.
