# Supplementary material for: Incorporating Artificial Intelligence in Qualitative Research: Exploring the Role of ChatGPT in Thematic Analysis
Source: Med Sci Educ. 2025 Dec 4;36(1):73–9. doi: 10.1007/s40670-025-02587-2 (PMC13043985; doi:10.1007/s40670-025-02587-2)
Supplement: Supplementary file 1 — (DOCX 15.4 KB) [file 40670_2025_2587_MOESM1_ESM.docx]

| Theme | Description |
| --- | --- |
| **Gratitude and Respect** | Students express deep appreciation for the opportunity to dissect cadavers and acknowledge the responsibility of treating donors with utmost respect. donated their bodies for educational purposes. donors' selflessness. |
| **Apprehension and Nervousness** | Students express apprehension about the emotional and practical aspects of dissection. |
| **Anticipation and Excitement** | Students express excitement about the educational journey and gaining a deeper understanding of the human body. |
| **Reflection on Life and Death** | Dissection prompts contemplation about the cycle of life, the value of human life, and the connection between life and death. Students contemplate the significance of the journey from birth to death and the role of dissection in continuing that journey in a different form. |
| **Historical Context** | Students reflect on the historical background of cadaveric dissection, recognizing past unethical practices and the need to approach it ethically and respectfully. Some students also acknowledging its problematic history. (any reference to Medical Apartheid). |
| **Culture Context** | References are made to cultural symbols to frame the significance of dissection. |
| **Learning Opportunity** | Students view dissection as a valuable learning experience that will contribute to their knowledge of human anatomy and personal and professional growth. Students acknowledge the impact that cadaveric dissection will have on their future medical practice, emphasizing the value of this learning method (impact on medical practice). |
| **Connection to Donors** | Students emphasize the importance of understanding and connecting with the individual donors, recognizing each one as a unique person with a life story. |
| **Empathy and Compassion** | Students aim to develop empathy and compassion for the donors and their families, acknowledging the selfless act of body donation. Dissection is seen as an opportunity to develop empathy by understanding the lives of donors. |
| **Symbolism** | Various symbols, such as trees, roots, and olive branches, are used to convey deeper meanings about dissection. |
| **Ethical Considerations** | Students consider the ethical aspects of using cadaveric donors and the importance of respecting their wishes, donor consent, and the history of body donations. |
| **Collaboration and Support** | Students recognize the role of collaboration and teamwork in the dissection process, often working in groups to learn from the donors. Students value the collaboration with peers and the emotional support they receive in the dissection process. |
| **Responsibility** | Students feel a sense of responsibility in handling the donated bodies and in their future medical practice. |
| **Physical and Sensory Experience** | Students anticipate the sensory aspects of dissection, like the smell and tactile sensations. |
